# Impact of reinforced pancreaticojejunostomy with or without tissue adhesive glue modified cyanoacrylate following pancreaticoduodenectomy: a randomized controlled clinical trial

**DOI:** 10.1186/s12957-026-04476-3

**Published:** 2026-06-30

**Authors:** Saleh Khairy Saleh, Mohamed Sadek Farahat, Tantawi Abdelnaeem Mohamed, Mina Makram Hendy

**Affiliations:** Department of Surgery, Faculty of Medicine, Minia University, Minia, Egypt

**Keywords:** Pancreaticoduodenectomy, Pancreaticojejunostomy, Postoperative pancreatic fistula, Cyanoacrylate, Glubran^®^2, Tissue adhesive, Randomized controlled trial

## Abstract

**Background:**

Postoperative pancreatic fistula (POPF) remains the most significant complication following pancreaticoduodenectomy (PD). This randomized controlled trial evaluated the efficacy of reinforcing the pancreaticojejunostomy (PJ) with Glubran^®^2, a modified N-butyl-2-cyanoacrylate adhesive, in reducing the rate of clinically relevant POPF.

**Methods:**

This single-center randomized controlled trial with blinded patients and blinded outcome assessors enrolled 100 consecutive patients undergoing open PD for pancreatic head or periampullary cancer between 15 January 2025 and 15 January 2026. Patients were randomized 1:1 to Glubran^®^2-reinforced PJ (Glubran^®^2 group) or conventional PJ (Control group). The primary endpoint was the rate of POPF (Grade B/C per ISGPS 2016) within the 90-day postoperative period. Operating surgeons were not blinded; ward physicians, outcome assessors and statisticians were blinded.

**Results:**

POPF (Grade B/C) occurred in 3 patients (6%) in the Glubran^®^2 group versus 11 patients (22%) in the Control group (*p* = 0.041; Fisher’s exact test). Grade B POPF was 4% versus 16% (*p* = 0.092) and Grade C POPF was 2% versus 6% (*p* = 0.617); individual-grade comparisons were prespecified as exploratory. Overall morbidity (any complication) was 18% versus 54% (*p* < 0.001) and severe complications (Clavien-Dindo ≥ IIIa) were 10% versus 36% (*p* = 0.004). The Glubran^®^2 group had earlier drain removal (median 6 [IQR 5–7] vs. 14 [IQR 9–18] days, *p* < 0.001) and shorter hospital stay (10.8 ± 3.2 vs. 19.2 ± 7.1 days, *p* < 0.001). On multivariable analysis, Glubran^®^2 use was an independent protective factor (adjusted OR 0.18, 95% CI 0.06–0.54, *p* = 0.002), after adjustment for soft pancreatic texture and main pancreatic duct ≤ 3 mm. The effect was consistent on per-protocol and surgeon-clustered sensitivity analyses.

**Conclusions:**

In this single-center randomized trial, reinforcement of pancreaticojejunostomy with Glubran^®^2 was associated with a significantly lower rate of POPF (Grade B/C), shorter time to drain removal, lower overall morbidity, and shorter length of hospital stay, with an acceptable safety profile. These hypothesis-generating findings warrant confirmation in an adequately sized, multicenter trial with operator-independent application before any recommendation for routine clinical adoption.

**Trial registration:**

ClinicalTrials.gov NCT06756074. Registered 24 December 2024 (prospective). https://clinicaltrials.gov/study/NCT06756074.

## Introduction

Pancreaticoduodenectomy (PD) is the only potentially curative treatment for malignancies of the pancreatic head and the periampullary region. Although operative mortality has fallen below 5% in high-volume centers, postoperative morbidity remains substantial, ranging from 30% to 50% [1]. The dominant driver of this morbidity is postoperative pancreatic fistula (POPF), the so-called “Achilles’ heel” of pancreatic surgery. POPF is associated with intra-abdominal abscess, sepsis, post-pancreatectomy hemorrhage, prolonged hospitalization, and death [2, 3].

A wide range of surgical techniques and pharmacological strategies has been investigated to prevent POPF, but no single intervention has been definitively superior [4]. Tissue adhesives, especially fibrin sealants, have been extensively evaluated; meta-analyses have not shown a consistent benefit, likely because fibrin is degraded by pancreatic proteolytic enzymes [5, 6]. By contrast, cyanoacrylate adhesives polymerize rapidly into a flexible waterproof film, possess high tensile strength, and resist enzymatic degradation [7].

Glubran^®^2 (GEM S.r.l., Viareggio, Italy) is a modified N-butyl-2-cyanoacrylate adhesive containing a methacryloxysulfolane co-monomer that lowers the polymerization temperature and reduces tissue toxicity while preserving adhesive strength [8]. To date, the only previous randomized trial specifically evaluating Glubran^®^2 reinforcement of pancreaticojejunostomy is the open-label trial by Gaspar et al. (*n* = 63) [9]; the remaining literature is observational. Further randomized evidence is therefore needed. We hypothesized that topical application of Glubran^®^2 to the pancreaticojejunal anastomosis would reduce the incidence of POPF by mechanically sealing the anastomotic line. The aim of this trial was to evaluate the impact of Glubran^®^2 reinforcement on POPF rate and clinical outcomes after open pancreaticoduodenectomy.

## Methods

### Study design and setting

**Fig. 1:**
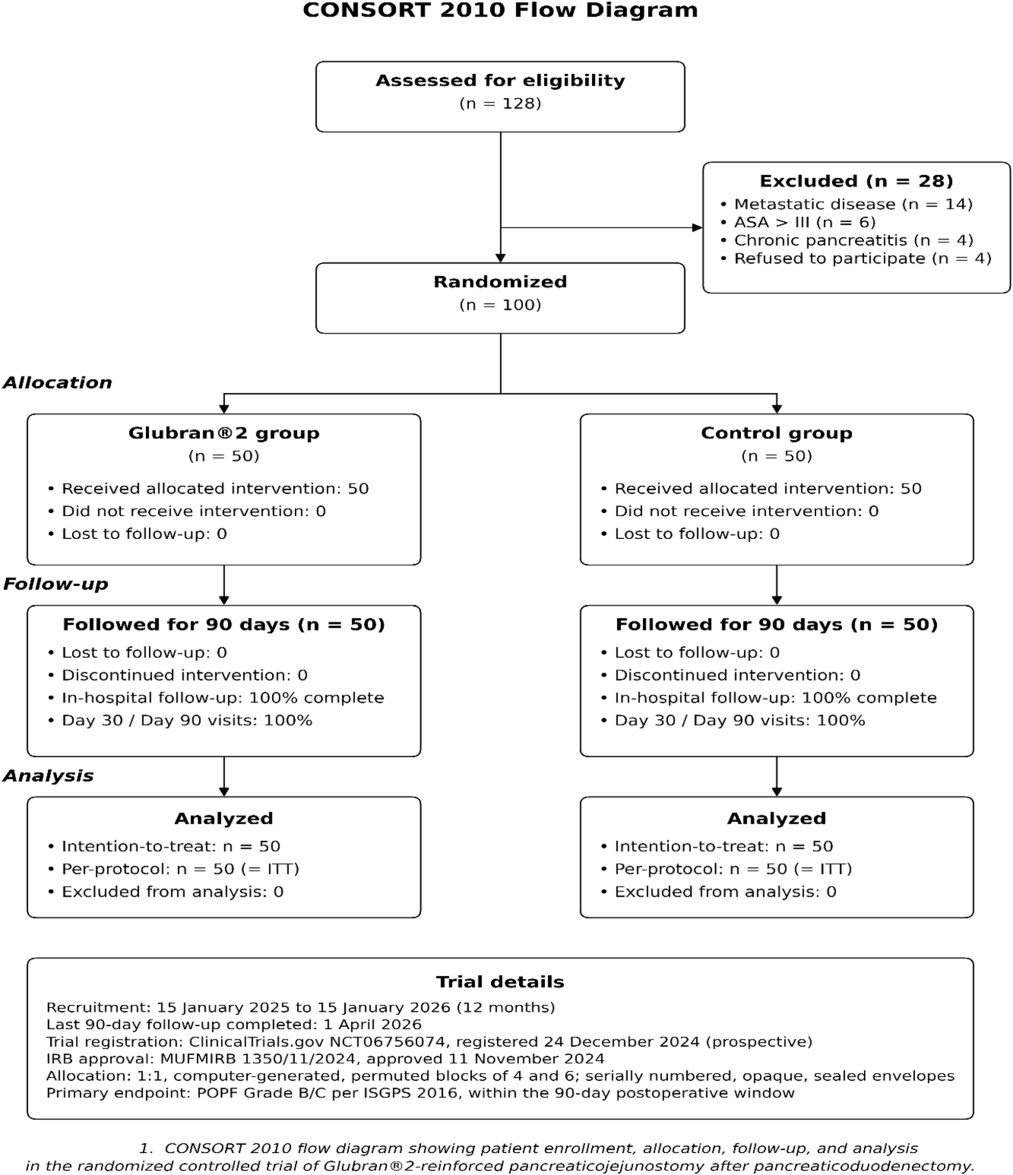
CONSORT 2010 flow diagram

This was a single-center, parallel-group, randomized controlled trial with 1:1 allocation conducted at the Liver and GIT Hospital, Faculty of Medicine, Minia University, Egypt. The trial protocol was approved by the Institutional Review Board of the Faculty of Medicine, Minia University (MUFMIRB, IRB number 1350/11/2024; approval date 11 November 2024) and prospectively registered at ClinicalTrials.gov (NCT06756074) on 24 December 2024. The first patient was randomized on 15 January 2025; the last on 15 January 2026; the 90-day follow-up of the last patient was completed on 1 April 2026. Recruitment lasted 12 months. The trial was conducted in accordance with the Declaration of Helsinki and is reported in line with the CONSORT 2010 statement (Fig. 1). Written informed consent was obtained from every participant before enrollment.

### Participants

#### Inclusion criteria

(1) adult patients aged 18–75 years; (2) elective open pancreaticoduodenectomy (classic Whipple or pylorus-preserving) for pancreatic head adenocarcinoma or periampullary malignancy (cholangiocarcinoma, ampullary carcinoma, duodenal carcinoma); (3) standard duct-to-mucosa pancreaticojejunostomy reconstruction planned; (4) ASA physical status I–III; (5) ECOG performance status 0–2; (6) adequate hepatic, renal, and bone-marrow function (total bilirubin < 3 × ULN, creatinine < 2.0 mg/dL, platelet count > 100,000/µL); (7) written informed consent.

#### Exclusion criteria

(1) ASA physical status > III; (2) distant metastasis or unresectable disease on preoperative imaging or at exploration; (3) chronic pancreatitis with calcifications; (4) previous pancreatic surgery; (5) emergency surgery; (6) planned pancreaticogastrostomy or Roux-en-Y reconstruction; (7) planned laparoscopic or robotic approach (excluded to standardize technique); (8) known cyanoacrylate hypersensitivity; (9) pregnancy or lactation; (10) refusal or inability to provide informed consent.

All consecutive patients meeting the eligibility criteria during the recruitment period were screened. Reasons for non-inclusion of screened patients are reported in the CONSORT diagram.

### Randomization, allocation concealment and blinding

Eligible patients were randomly assigned 1:1 to Glubran^®^2 reinforcement or control using a computer-generated randomization sequence with permuted blocks of 4 and 6 prepared by an independent statistician. Allocation was concealed by serially numbered, opaque, sealed envelopes. Each envelope was opened in the operating theatre by the scrub nurse or anesthesiologist after completion of the pancreaticojejunostomy and before abdominal closure.

Blinding is reported following the recommendations of Probst et al. [10]: patients, blinded; operating surgeons, not blinded (intervention visible); anaesthesiologists / scrub nurses, not blinded (opened the envelope); surgical residents and ward physicians responsible for routine postoperative orders, drain management, drain removal, imaging requests and discharge, blinded (the group label was not entered in the patient chart); outcome assessors, blinded; data collectors, blinded; statistical analysts, blinded (the allocation list was unmasked only after database lock).

Decisions on reoperation, percutaneous drainage and radiologic intervention were made by an unblinded duty surgeon when clinically required and were always reviewed by a second blinded senior consultant for confirmation. Residual performance bias from unblinded operating surgeons cannot be excluded and is acknowledged in the Limitations.

### Surgical technique

All operations were performed by three experienced consultant hepatopancreatobiliary surgeons, each contributing similar numbers of cases to both arms (S.K.S. 22 vs. 21; M.S.F. 14 vs. 15; T.A.M. 14 vs. 14, Glubran^®^2 vs. Control). The randomization sequence was generated independently of surgeon identity. A standardized open Whipple or pylorus-preserving pancreaticoduodenectomy was performed depending on tumor location and oncological margins. Reconstruction used a single retrocolic loop of jejunum. The pancreaticojejunostomy was constructed as an end-to-side, two-layer duct-to-mucosa anastomosis: the outer layer was 4 − 0 polypropylene interrupted sutures (8–12 sutures) approximating the pancreatic capsule to the jejunal seromuscular layer; the inner layer was 5 − 0 polydioxanone interrupted sutures (6–8 sutures) approximating the pancreatic duct mucosa to the jejunal mucosa. No internal pancreatic duct stent was used.

Hepaticojejunostomy (4 − 0 polydioxanone, continuous or interrupted depending on bile-duct caliber) and gastrojejunostomy or duodenojejunostomy (4 − 0 polypropylene, continuous) were then performed identically in both arms.

In the Glubran^®^2 group, after completion of the entire pancreaticojejunal anastomosis (and subsequent hepaticojejunostomy and gastrojejunostomy/duodenojejunostomy), final intraoperative hemostasis was confirmed and, immediately before abdominal wall closure, the pancreaticojejunostomy area was gently dried with sterile gauze swabs. Glubran^®^2 (N-butyl-2-cyanoacrylate with methacryloxysulfolane; GEM S.r.l., Viareggio, Italy) was then applied topically as follows: 1 mL of the material was administered via a Glubran^®^2 micro-drop apparatus (GEM). To bring the posterior aspect of the anastomosis into view, the pancreatic stump and the adjacent jejunal loop were gently mobilized with a moistened gauze pack and atraumatic forceps. The adhesive was then delivered drop-by-drop in a continuous sweeping motion along the visible suture line, covering the entire anterior surface and the directly visualized portion of the posterior surface of the pancreaticojejunal anastomosis. The most medial aspect, adjacent to the portal vein / superior mesenteric vein (PV/SMV) confluence, could not be safely visualized and was deliberately left uncoated to avoid inadvertent glue deposition on the venous structures. The polymerization time was approximately 60–90 s, forming a flexible, waterproof film over the suture line, which was visually verified by the operating surgeon as a thin transparent-to-whitish film overlying the visible circumference.

**Fig. 2:**
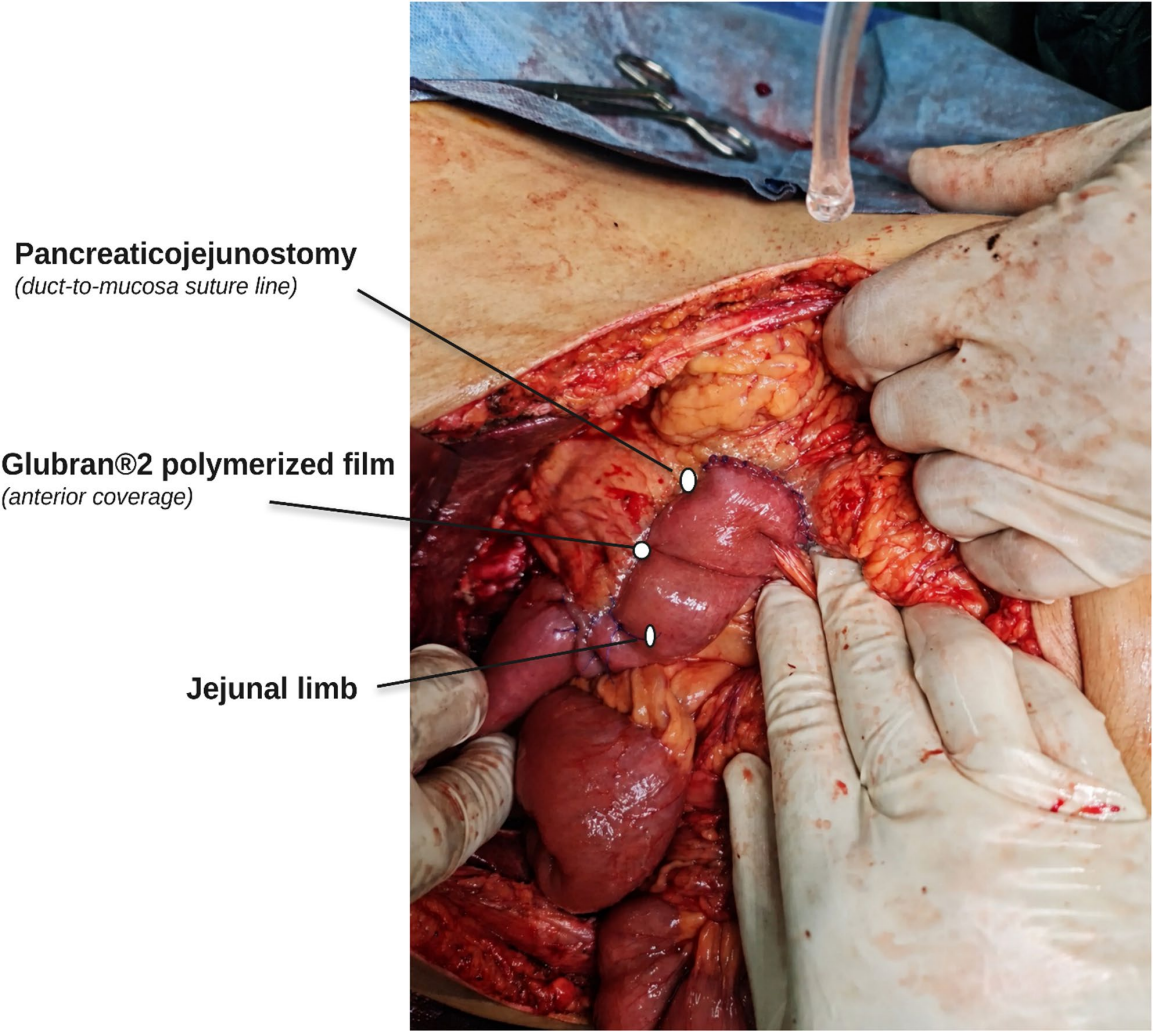
Intraoperative photograph showing application of Glubran^®^2 to the pancreaticojejunostomy

**Fig. 3:**
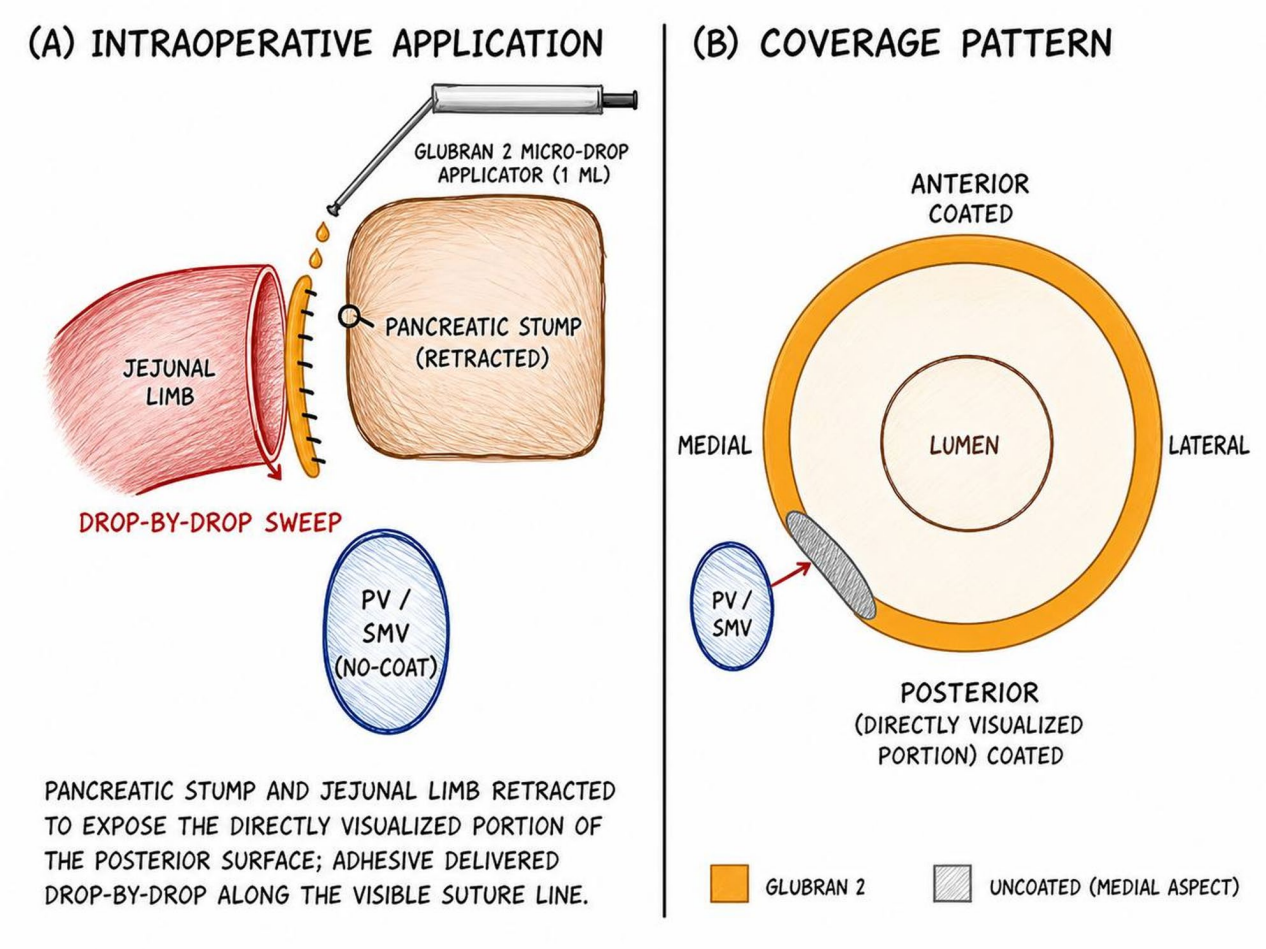
Diagram showing application technique of Glubran^®^2 over the pancreaticojejunal anastomosis. **A** After completion of the pancreaticojejunal anastomosis, 1 mL of Glubran®2 is applied drop-by-drop along the visible posterior suture line with the dedicated micro-drop applicator. The medial portion adjacent to the portal vein / superior mesenteric vein (PV/SMV) is left uncoated. **B** Axial cross-section showing the resulting coverage: the entire anterior surface and the directly visualized portion of the posterior surface are coated; the medial-posterior portion adjacent to the PV/SMV is left uncoated

The pancreaticoduodenectomies themselves were distributed across the three consultant surgeons (as above); to remove application-technique heterogeneity from the evaluation of the adhesive, the brief topical-application step was standardized using a written application protocol and a laminated step-by-step card. The corresponding author (S.K.S.) personally applied the glue in 47 of the 50 cases and directly supervised the other 3 (performed by another consultant after a documented training period). This was a deliberate design choice intended to maximize internal validity by isolating the biological effect of the adhesive from inter-operator variability in its application; the consequent limitation for generalizability is addressed explicitly in the Limitations. An intraoperative photograph is shown in Fig. 2. A schematic diagram of the application technique is shown in Fig. 3.

In the Control group, no tissue adhesive was used. After completion of all anastomoses, hemostasis and warm-saline lavage, two closed-suction Blake drains (19 Fr) were placed in all patients (one posterior to the pancreaticojejunostomy, one near the hepaticojejunostomy) and exited via separate stab incisions in the right upper quadrant. Concurrent venous resection (portal/superior mesenteric vein wedge or segmental resection with reconstruction) was performed when oncologically required; no arterial resection was performed. Glubran^®^2 was not applied to any vascular suture line.

### Drain management

The drain-removal protocol was identical in both arms and was applied by the blinded ward team. Drains were removed when ALL three of the following conditions were met simultaneously: (i) drain fluid amylase (DFA) less than 3 × the upper limit of normal of serum amylase (i.e. < 600 U/L assuming a serum ULN of 200 U/L); (ii) daily drain output < 50 mL on two consecutive days; and (iii) absence of clinical features of POPF (no fever, no abdominal pain, no purulent drainage). No drain was removed before postoperative day 5. The two drains were assessed and removed independently. The hepaticojejunostomy drain was generally removed first, followed by the pancreaticojejunostomy drain; the “drain removal time” reported in the Results corresponds to the day of removal of the last (i.e. PJ) drain.

### Perioperative management

Perioperative care followed a standardized institutional pathway. Prophylactic antibiotic (ceftriaxone 2 g intravenously) was administered at induction and continued for 48 h. No patient received prophylactic somatostatin or octreotide. All patients received a nasogastric tube (removed when output < 200 mL/day and bowel sounds were present, typically POD 3–5), a nasoenteral feeding tube placed distal to the gastrojejunostomy (early enteral nutrition started within 24–48 h), and an indwelling urinary catheter (removed POD 1–2). Drain fluid amylase was measured on POD 1, 3, 5 and 7, with serum amylase and lipase measured concurrently. Therapeutic octreotide (100 µg s.c. three times daily) was administered when clinically required for Grade B POPF management, by blinded ward physicians, applying the same indications in both arms.

### Outcomes and definitions

All outcomes followed published international definitions: POPF — ISGPS 2016 [2]; post-pancreatectomy hemorrhage — ISGPS [3]; delayed gastric emptying — ISGPS (Wente et al.) [11]; surgical-site infection — CDC [12]; surgical complications — Clavien-Dindo classification [13]; Comprehensive Complication Index — CCI [www.assessurgery.com].

#### Primary endpoint

rate of POPF (Grade B or Grade C per ISGPS 2016) within the 90-day postoperative window. Grade B requires deviation from the normal postoperative pathway (drain in situ for > 3 weeks, percutaneous drainage, endoscopic or angiographic intervention, parenteral or enteral nutrition support, therapeutic octreotide administration, or use of antibiotics specifically for fistula-related infection). Grade C requires reoperation, single- or multi-organ failure with ICU management, or death attributable to POPF. Biochemical leak (drain fluid amylase > 3 × ULN of serum amylase without clinical sequelae) is not classified as POPF in ISGPS 2016 and was reported separately as an exploratory outcome.

#### Secondary endpoints

post-pancreatectomy hemorrhage; delayed gastric emptying; biliary fistula; intra-abdominal abscess; surgical-site infection; reoperation; readmission within 30 days; drain removal time; length of hospital stay; ICU stay; 90-day mortality; overall morbidity (any complication); severe morbidity (Clavien-Dindo ≥ IIIa); and Comprehensive Complication Index.

#### Exploratory outcomes

biochemical leak rate; composite drain-amylase outcome (BL + POPF); per-drain DFA values on POD 1 and 3.

### Statistical analysis and sample sizesize

#### Sample-size derivation

 The primary endpoint was clinically relevant POPF (CR-POPF, Grade B/C per ISGPS 2016); biochemical leak (former Grade A) was never used as the primary endpoint. For sample-size estimation we anchored the calculation on a prespecified composite anastomotic-leak control rate, defined as the sum of biochemical leak and CR-POPF — that is, the total drain-amylase–positive leak of any grade. This composite was selected because, at the time of protocol design, it represented the most stable and directly measurable institutional benchmark. In our retrospective audit of 86 consecutive PDs (2021–2023), the total anastomotic-leak rate (ISGPS 2005, all grades) was 41/86 (47.7%), of which CR-POPF (Grade B/C) accounted for 22.1%; we therefore adopted a deliberately conservative composite control rate of 44%. The assumed intervention (composite) rate of 19% — a 25-percentage-point absolute reduction — was derived from the best cyanoacrylate-specific evidence available at protocol design (Barakat et al., topical 2-octyl-cyanoacrylate [14]), because no completed randomized trial of Glubran^®^2 reinforcement of pancreaticojejunostomy then existed (the trial by Gaspar et al. [9] was published in 2025, after our protocol had been registered). With α = 0.05 (two-sided) and 80% power, 50 patients per group (*n* = 100) were required. The prespecified composite endpoint is reported in Table 3. The observed composite reduction (34% to 8%) was larger than the 44%-to-19% reduction assumed at design, indicating that the planning assumptions were conservative rather than optimistic.

#### Power and precision

 The trial reached its prespecified sample size of 50 patients per group and detected a statistically significant difference on the primary endpoint (CR-POPF Grade B/C, *p* = 0.041, Fisher’s exact test). For the prespecified composite leak endpoint on which the calculation was anchored, the design provided in excess of 95% power against the observed event rates. In keeping with current statistical guidance, observed (post-hoc) power computed from the realised result is uninformative once a significant effect has been demonstrated, and is therefore not reported; instead, every effect estimate is accompanied by a 95% confidence interval to convey precision. Low-frequency individual-grade comparisons (e.g. Grade C alone) and stratified subgroup analyses were prespecified as exploratory and are interpreted descriptively, with confidence intervals, rather than as confirmatory hypothesis tests.

#### Analysis populations

 The intention-to-treat (ITT) population included all 100 randomized patients in their assigned groups. All 100 patients received their allocated intervention as protocolized; the per-protocol (PP) population was therefore identical to the ITT population (*n* = 50 vs. 50).

#### Statistical methods

Categorical variables are reported as n (%) and compared using Fisher’s exact test (used when any expected cell count was < 5) or the χ² test. All categorical p-values reported in this revision were recomputed and verified with the two-sided Fisher’s exact test. Continuous variables that were approximately normally distributed (operative time, blood loss, length of stay) are reported as mean ± SD and compared with the independent-samples t-test; non-normal continuous variables (drain fluid amylase, drain removal time, Comprehensive Complication Index) are reported as median [IQR] and compared with the Mann-Whitney U test. Univariable and multivariable logistic regression were used to identify independent predictors of POPF. To respect the events-per-variable rule with 14 POPF events, the multivariable model included only three pre-specified clinically relevant variables: Glubran^®^2 use, soft pancreatic texture, and main pancreatic duct ≤ 3 mm. Results are presented as odds ratios with 95% confidence intervals. To assess robustness we also performed: (i) a per-protocol sensitivity analysis; (ii) a sensitivity analysis excluding patients with positive bile cultures; and (iii) a generalized estimating equation accounting for surgeon-level clustering. Only the primary endpoint was tested confirmatorily at α = 0.05; secondary and exploratory outcomes are reported as point estimates with 95% confidence intervals and unadjusted p-values and were also examined with Benjamini-Hochberg false-discovery-rate adjustment. A p-value < 0.05 was considered statistically significant for the primary endpoint. Analyses were performed in SPSS 26.0 (IBM Corp., Armonk, NY).

## Results

### Patient flow

During the recruitment period, 128 patients were screened for eligibility. Twenty-eight patients were excluded prior to randomization: metastatic disease (*n* = 14), ASA > III (*n* = 6), chronic pancreatitis (*n* = 4), and refusal to participate (*n* = 4). One hundred patients were randomized (50 to each arm) and all 100 received their allocated intervention as protocolized. There was no loss to follow-up; all 100 patients completed the 90-day follow-up. The CONSORT diagram is shown as Fig. 4.

### Baseline characteristics

The two groups were well balanced for demographic, clinical and oncological characteristics, including age, sex, BMI, ASA status, diabetes, preoperative jaundice, preoperative biliary drainage, serum albumin, neoadjuvant therapy and tumor type (Table 1). No patient received neoadjuvant radiotherapy.

**Table 1:** Baseline demographic, clinical and oncological characteristics

Characteristic	Glubran^®^2 (*n* = 50)	Control (*n* = 50)	*p*-value
Age, years, mean ± SD (range)	58.4 ± 10.2 (39–74)	59.1 ± 11.5 (37–75)	0.745
Male, n (%)	28 (56%)	26 (52%)	0.689
BMI, kg/m², mean ± SD (range)	24.2 ± 3.5 (18.1–32.4)	24.8 ± 3.1 (18.6–31.7)	0.372
ASA II/III, n (%)	42 (84%)	40 (80%)	0.603
Diabetes mellitus, n (%)	12 (24%)	14 (28%)	0.648
Preoperative jaundice, n (%)	25 (50%)	28 (56%)	0.548
Preoperative biliary drainage, n (%)	18 (36%)	20 (40%)	0.677
Serum albumin, g/dL, mean ± SD (range)	3.6 ± 0.5 (2.6–4.5)	3.5 ± 0.6 (2.4–4.6)	0.384
Neoadjuvant chemotherapy, n (%)	6 (12%)	5 (10%)	0.749
Neoadjuvant radiotherapy, n (%)	0 (0%)	0 (0%)	—
Pancreatic head adenocarcinoma, n (%)	24 (48%)	26 (52%)	0.892
Ampullary carcinoma, n (%)	14 (28%)	12 (24%)	
Cholangiocarcinoma, n (%)	8 (16%)	7 (14%)	
Duodenal adenocarcinoma, n (%)	4 (8%)	5 (10%)	

### Intraoperative data and fistula-risk profile

Operative time and estimated blood loss were comparable between groups (Table 2). Pancreatic texture and main pancreatic duct diameter were balanced. The original Fistula Risk Score (Callery 2013) and the alternative FRS (a-FRS, Mungroop 2019) were calculated for every patient and were balanced between groups. Concurrent venous resection was performed in 4 (8%) and 5 (10%) patients in the Glubran^®^2 and Control groups respectively (*p* = 1.000); no arterial resection was performed.

**Table 2:** Intraoperative data and fistula risk profile

Parameter	Glubran^®^2 (*n* = 50)	Control (*n* = 50)	*p*-value
Operative time, min, mean ± SD	345 ± 48	338 ± 52	0.485
Estimated blood loss, mL, mean ± SD	450 ± 120	475 ± 140	0.354
Soft pancreatic texture, n (%)	28 (56%)	30 (60%)	0.683
Main pancreatic duct ≤ 3 mm, n (%)	32 (64%)	34 (68%)	0.670
Positive bile culture, n (%)	22 (44%)	25 (50%)	0.548
Concurrent venous resection, n (%)	4 (8%)	5 (10%)	1.000
a-FRS — negligible/low, n (%)	12 (24%)	11 (22%)	0.962
a-FRS — moderate, n (%)	22 (44%)	23 (46%)	
a-FRS — high, n (%)	16 (32%)	16 (32%)	
Original FRS (Callery), median [IQR]	5 [3–7]	5 [3–7]	0.812
Pancreatic resection margin positive (R1), n (%)	4 (8%)	5 (10%)	0.727

### Primary endpoint: postoperative pancreatic fistula

POPF (Grade B/C) — the primary endpoint — occurred in 3 patients (6%) in the Glubran^®^2 group versus 11 patients (22%) in the Control group (*p* = 0.041, Fisher’s exact test). Grade B POPF was 4% versus 16% (*p* = 0.092) and Grade C POPF was 2% versus 6% (*p* = 0.617); these individual-grade comparisons were prespecified as exploratory and are reported descriptively. The per-protocol analysis was identical to the ITT analysis (no protocol deviations in either arm). The 30-day and 90-day cumulative POPF (Grade B/C) rates are shown in Table 3. All POPF events were diagnosed within the initial 2-week intensive surveillance window; no new clinically relevant POPF cases were identified between POD 14 and POD 90 in either group. Biochemical leak (former Grade A under ISGPS 2005, no longer POPF under ISGPS 2016) — reported here as an exploratory outcome — occurred in 1 (2%) versus 6 (12%) patients (*p* = 0.112). The composite drain-amylase outcome (BL + POPF) — the prespecified sizing endpoint — was 4 (8%) versus 17 (34%) (*p* = 0.003).

In a stratified subgroup analysis by a-FRS category (Table 4), the protective effect of Glubran^®^2 on POPF was greatest in the moderate-risk (POPF 4.5% vs. 26.1%) and high-risk (12.5% vs. 31.3%) strata. Sensitivity analyses excluding patients with positive bile cultures (POPF 1/26 vs. 6/27, *p* = 0.100) and a generalized estimating equation accounting for surgeon-level clustering (surgeon effect non-significant, *p* = 0.41) were consistent with the primary result. Benjamini-Hochberg-adjusted q-values for the secondary outcomes did not change the qualitative conclusions.

**Table 3:** Pancreatic anastomotic outcomes

Outcome	Glubran^®^2 (*n* = 50)	Control (*n* = 50)	*p*-value
POPF Grade B/C (primary endpoint, 90-day)	3 (6%)	11 (22%)	0.041
• Grade B	2 (4%)	8 (16%)	0.092
• Grade C	1 (2%)	3 (6%)	0.617
POPF cumulative incidence at 30 days	3 (6%)	10 (20%)	0.071
POPF cumulative incidence at 90 days	3 (6%)	11 (22%)	0.041
Per-protocol POPF Grade B/C (PP = ITT)	3/50 (6%)	11/50 (22%)	0.041
Biochemical leak (former Grade A; exploratory)	1 (2%)	6 (12%)	0.112
Composite drain-amylase outcome (BL + POPF; exploratory)	4 (8%)	17 (34%)	0.003

*P-values* are from the two-sided Fisher’s exact test. The primary endpoint (CR-POPF Grade B/C) was the only confirmatory comparison; all other rows are secondary or prespecified exploratory outcomes reported with unadjusted p-values and interpreted descriptively. Benjamini–Hochberg false-discovery-rate adjustment of the secondary and exploratory comparisons did not alter the qualitative conclusions

**Table 4:** POPF (Grade B/C) stratified by a-FRS category

a-FRS category	Glubran^®^2 POPF, *n*/*N* (%)	Control POPF, *n*/*N* (%)	*p*-value
Negligible / Low	0/12 (0%)	0/11 (0%)	1.000
Moderate	1/22 (4.5%)	6/23 (26.1%)	0.096
High	2/16 (12.5%)	5/16 (31.3%)	0.394

### Postoperative complications and morbidity

Postoperative complications are summarized in Table 5. Overall morbidity (any complication) was 18% in the Glubran^®^2 group versus 54% in the Control group (*p* < 0.001). Severe complications (Clavien-Dindo ≥ IIIa) were 10% versus 36% (*p* = 0.004). The Comprehensive Complication Index was significantly lower in the Glubran^®^2 group (median 0 [IQR 0–20.9] vs. 26.2 [IQR 8.7–42.4], *p* < 0.001). For individual complications, intra-abdominal abscess showed a trend toward reduction (4% vs. 16%, *p* = 0.092), as did delayed gastric emptying (6% vs. 18%, *p* = 0.121), post-pancreatectomy hemorrhage (4% vs. 12%, *p* = 0.269), biliary fistula (2% vs. 8%, *p* = 0.362), surgical-site infection (6% vs. 16%, *p* = 0.200) and reoperation (2% vs. 10%, *p* = 0.204). Ninety-day mortality was 2% in the Glubran^®^2 group (1 case; respiratory failure in a patient with Grade C POPF) versus 4% in the Control group (2 cases; one Grade C POPF, one cardiac event), *p* = 1.000.

**Table 5:** Postoperative complications, Clavien-Dindo classification and Comprehensive Complication Index

Outcome	Glubran^®^2 (*n* = 50)	Control (*n* = 50)	*p*-value
Post-pancreatectomy hemorrhage, n (%)	2 (4%)	6 (12%)	0.269
Delayed gastric emptying, n (%)	3 (6%)	9 (18%)	0.121
Biliary fistula, n (%)	1 (2%)	4 (8%)	0.362
Intra-abdominal abscess, n (%)	2 (4%)	8 (16%)	0.092
Surgical-site infection, n (%)	3 (6%)	8 (16%)	0.200
Reoperation, n (%)	1 (2%)	5 (10%)	0.204
90-day mortality, n (%)	1 (2%)	2 (4%)	1.000
Clavien-Dindo Grade I, n (%)	0 (0%)	2 (4%)	0.495
Clavien-Dindo Grade II, n (%)	4 (8%)	7 (14%)	0.525
Clavien-Dindo Grade IIIa, n (%)	3 (6%)	11 (22%)	0.041
Clavien-Dindo Grade IIIb, n (%)	1 (2%)	5 (10%)	0.204
Clavien-Dindo Grade IV, n (%)	0 (0%)	0 (0%)	1.000
Clavien-Dindo Grade V, n (%)	1 (2%)	2 (4%)	1.000
Overall morbidity (any complication), n (%)	9 (18%)	27 (54%)	< 0.001
Severe complications (≥ IIIa), n (%)	5 (10%)	18 (36%)	0.004
Comprehensive Complication Index, median [IQR]	0 [0–20.9]	26.2 [8.7–42.4]	< 0.001

*P-values *are from the two-sided Fisher’s exact test for low-count categorical comparisons and from the χ² test where all expected cell counts were ≥ 5 (overall morbidity, severe complications). These are secondary outcomes reported with unadjusted p-values; Benjamini–Hochberg adjustment did not alter the qualitative conclusions for overall morbidity and severe complications

### Recovery parameters and drain fluid amylase

Recovery parameters are presented in Table 6. Drain fluid amylase values were strongly non-normal and are therefore reported as median [IQR] with Mann-Whitney U-test comparison. The PJ-drain DFA on POD 1 was substantially lower in the Glubran^®^2 group (median 620 [IQR 380–1,150] vs. 3,200 [1,400–6,800] U/L, *p* < 0.001) and remained lower on POD 3. The HJ-drain DFA was likewise lower on POD 1 (95 [50–180] vs. 350 [120–820] U/L, *p* = 0.001), reflecting reduced anastomotic leakage. Drain removal occurred earlier in the Glubran^®^2 group (median 6 [IQR 5–7] days vs. 14 [9, 14–22] days, *p* < 0.001) and length of hospital stay was shorter (10.8 ± 3.2 vs. 19.2 ± 7.1 days, *p* < 0.001). The 30-day readmission rate was 4% versus 14% (*p* = 0.160).

**Table 6:** Postoperative recovery parameters and drain fluid amylase

Parameter	Glubran^®^2 (*n* = 50)	Control (*n* = 50)	*p*-value
PJ-drain DFA POD 1, U/L, median [IQR]	620 [380–1,150]	3,200 [1,400–6,800]	< 0.001
PJ-drain DFA POD 3, U/L, median [IQR]	240 [120–490]	1,850 [620–4,100]	< 0.001
HJ-drain DFA POD 1, U/L, median [IQR]	95 [50–180]	350 [120–820]	0.001
Drain removal time, days, median [IQR]	6 [5–7]	14 [9–18]	< 0.001
Length of hospital stay, days, mean ± SD	10.8 ± 3.2	19.2 ± 7.1	< 0.001
Readmission within 30 days, n (%)	2 (4%)	7 (14%)	0.160

### Risk-factor analysis

On multivariable logistic regression with three pre-specified clinically relevant covariates, Glubran^®^2 use was an independent protective factor against POPF (adjusted OR 0.18, 95% CI 0.06–0.54, *p* = 0.002), after adjustment for soft pancreatic texture (adjusted OR 4.25, 95% CI 1.52–11.8, *p* = 0.006) and main pancreatic duct ≤ 3 mm (adjusted OR 3.15, 95% CI 1.12–8.85, *p* = 0.029) (Table 7). Univariable associations for the remaining variables (age > 65, BMI > 25, operative time > 360 min, blood loss > 500 mL) are reported in Table 6 but were not entered into the multivariable model.

**Table 7:** Univariable and multivariable analysis of risk factors for POPF

Variable	Univariable OR (95% CI)	Univariable *p*	Multivariable OR (95% CI)	Multivariable *p*
Glubran^®^2 use (vs. Control)	0.17 (0.05–0.55)	0.001	0.18 (0.06–0.54)	0.002
Soft pancreatic texture	3.94 (1.22–12.76)	0.004	4.25 (1.52–11.80)	0.006
Main pancreatic duct ≤ 3 mm	3.88 (1.05–14.26)	0.012	3.15 (1.12–8.85)	0.029
Age > 65 years	1.19 (0.44–3.21)	0.452	—	—
BMI > 25 kg/m²	2.18 (0.82–5.78)	0.125	—	—
Operative time > 360 min	1.46 (0.55–3.82)	0.342	—	—
Blood loss > 500 mL	1.90 (0.72–5.01)	0.215	—	—

## Discussion

### Principal findings

In this single-center randomized controlled trial of 100 patients undergoing open pancreaticoduodenectomy, reinforcement of the pancreaticojejunostomy with Glubran^®^2 was associated with a significant reduction in POPF (Grade B/C), a lower Comprehensive Complication Index, fewer severe complications, earlier drain removal and shorter hospital stay. Glubran^®^2 use remained an independent protective factor on multivariable analysis after adjustment for the dominant intrinsic risk factors (soft pancreatic texture and small main pancreatic duct), and the effect was consistent across per-protocol, surgeon-clustered, and bile-culture-stratified sensitivity analyses.

The reduction in clinically relevant POPF was concentrated in the Grade B category. Although the trial was sized for the composite Grade B/C primary endpoint and individual-grade comparisons were prespecified as exploratory, this pattern is biologically plausible. Grade B fistulas characteristically arise from sustained low-flow pancreatic-juice extravasation through small needle-hole defects rather than from frank anastomotic dehiscence. Glubran^®^2, by polymerizing rapidly into a flexible waterproof film mechanically interdigitating the suture line, is well suited to sealing exactly this type of low-pressure micro-leak; it is unlikely to compensate for true dehiscence (the substrate of Grade C). The smaller absolute reduction observed for Grade C is consistent with this pathophysiology and with the low frequency of the Grade C event.

### Reduction in overall morbidity and complications

The protective effect extended beyond POPF to overall morbidity, severe complications (Clavien-Dindo ≥ IIIa), and the Comprehensive Complication Index. Individual complications recognized as downstream consequences of pancreatic leak — intra-abdominal abscess, delayed gastric emptying, post-pancreatectomy hemorrhage, biliary fistula, surgical-site infection, and reoperation — all showed trends toward reduction, although individual differences did not consistently reach statistical significance, reflecting the limited frequency of these low-incidence outcomes. The aggregate signal across multiple downstream complications is consistent with a single unifying mechanism: prevention of pancreatic juice leakage at the index anastomosis attenuates the cascade of secondary septic and inflammatory complications.

### Impact on recovery parameters and hospital stay

Drain fluid amylase was significantly lower in the Glubran^®^2 group from postoperative day 1 onward, indicating immediate anastomotic integrity rather than delayed sealing. The early DFA elevation is a recognized prognostic marker for POPF [23, 24]; its attenuation in the intervention group is consistent with the proposed mechanical-sealing mechanism. Earlier drain removal and shorter hospital stay followed, with implications for patient experience and healthcare resource utilization. The 30-day readmission rate showed a non-significant trend toward reduction, suggesting that the benefit may extend beyond the index hospitalization, although confirmation requires a larger sample.

### Mechanism of action

N-butyl-2-cyanoacrylate polymerizes within 60–90 s on contact with anionic tissue fluids, forming a solid elastic film that seals suture-line needle holes and the microscopic gap between the pancreatic parenchyma and the jejunal serosa [7, 16]. The polymer has high tensile strength and remains adherent during physiological tissue motion [25]. The methacryloxysulfolane co-monomer in Glubran^®^2 attenuates the exothermic polymerization reaction, reducing thermal damage and cytotoxicity compared with first-generation cyanoacrylates [8, 26]. Crucially, unlike fibrin sealants — which are protein substrates rapidly degraded by pancreatic proteases — cyanoacrylate is a synthetic polymer that resists enzymatic degradation and maintains mechanical integrity through the critical early healing window [17, 25]. This property explains why fibrin-sealant trials in pancreatic surgery have been largely negative [5, 18, 27] whereas cyanoacrylate-based reinforcement appears beneficial.

### Comparison with the existing evidence base

Our findings are consistent with the only previous randomized trial of Glubran^®^2 reinforcement of pancreaticojejunostomy (Gaspar et al., *n* = 63), which observed a reduction in POPF from 39.7% to 16.7% (*p* = 0.046) [9]. They are also directionally consistent with Barakat et al. (2-octyl-cyanoacrylate, observational) [14] and with Inthasotti et al. (N-butyl-2-cyanoacrylate, retrospective; non-significant trend) [21]. The ISGPS Evidence Map of Pancreatic Surgery — a living systematic review with meta-analyses [28] — classifies the evidence for fibrin-based pancreatic-anastomotic sealants as showing no benefit, while the evidence for cyanoacrylate-based sealants remains sparser; our trial therefore adds randomized evidence to a previously under-evidenced node of the map.

### Periampullary cancer subtypes

The five periampullary cancers — pancreatic ductal adenocarcinoma, distal cholangiocarcinoma, ampullary, duodenal and acinar-cell — differ substantially in tumor biology, in surgical complexity and in the anatomy of the pancreatic stump [29]. Ampullary, duodenal and distal bile-duct cancers more commonly present with a soft pancreas and a small main pancreatic duct — exactly the high-risk anatomical substrate in which Glubran^®^2 may confer the greatest absolute benefit. In our cohort, all four common periampullary subtypes were represented, and the protective effect of Glubran^®^2 was directionally consistent across subtypes (post-hoc analysis; exploratory only).

### Risk stratification and the protective effect of Glubran^®^2

On multivariable analysis Glubran^®^2 use was independently protective after adjustment for soft pancreatic texture and main pancreatic duct ≤ 3 mm — the two dominant intrinsic risk factors recognized in both the Callery FRS [30] and the alternative FRS [31]. BMI > 25 was associated with POPF on univariable but not multivariable analysis; this is consistent with BMI exerting its effect predominantly through pancreatic texture (steatotic pancreas) [32], such that adjustment for texture renders BMI non-significant. The subgroup analysis stratified by a-FRS category suggests that the protective effect of Glubran^®^2 was greatest in the moderate- and high-risk strata, supporting the clinical hypothesis that Glubran^®^2 acts by mitigating the risk inherent to unfavorable anatomy.

### Generalizability and external validity

The Liver and GIT Hospital, Minia University, performs 60–80 pancreaticoduodenectomies per year (intermediate- to high-volume by international classification). Our case-mix differs from many Western series in two relevant respects: a high prevalence of soft pancreas (60%) and a high prevalence of small main pancreatic duct (68%), reflecting a population dominated by ampullary and bile-duct cancers and a low background prevalence of long-standing chronic pancreatitis. The 22% control-group POPF rate is at the upper end of the contemporary international 10–22% range. The absolute risk reduction observed in this trial may therefore be smaller in lower-risk settings, although the relative risk reduction and the multivariable estimate suggest a real biological effect that should still be demonstrable in such settings. External multicenter validation is essential before generalization.

### Cost-effectiveness

We did not collect prospective cost data and have therefore not performed a formal cost-effectiveness analysis. The shorter hospital stay observed in the Glubran^®^2 group would be expected to translate into lower direct hospitalization costs; the formal cost-effectiveness analysis published by Gaspar et al. [9], in which Glubran^®^2 was a dominant strategy (incremental cost-effectiveness ratio of − US$35.65 per patient), is consistent with this expectation. A formal cost-effectiveness analysis in our healthcare setting is identified as a research priority.

### Safety

No adverse event was attributable to Glubran^®^2 itself in our cohort. There were no cases of bowel obstruction, abscess formation around residual adhesive, allergic reaction, or vascular complication in the four patients who underwent concurrent venous reconstruction. The biocompatibility and safety profile of Glubran^®^2 in cardiovascular, thoracic and neurosurgical applications [26, 33] is consistent with our experience. Late biodegradation, foreign-body granuloma and chronic peri-anastomotic inflammation were not specifically evaluated and warrant prospective study.

## Limitations

Our trial has several limitations. First, it is single-center and the case-mix is enriched for high-risk anatomy; external multicenter validation in different patient populations is essential. Second, although outcome assessment was blinded, the operating surgeons could not be blinded; residual performance bias cannot be excluded. Third, follow-up was limited to 90 days; anastomotic stricture, late glue-related complications, and pancreatic exocrine and endocrine function were not evaluated. Fourth, no formal cost-effectiveness analysis was performed. Fifth, in 47 of the 50 Glubran^®^2 cases the glue was applied by a single operator (S.K.S.) and under his direct supervision in the remaining 3; the observed effect therefore cannot be fully separated from the skill and experience of one surgeon, which constitutes a potential performance bias and limits external validity until the technique is shown to be reproducible by other surgeons after appropriate training. Sixth, the trial was sized for the composite Grade B/C primary endpoint and was anchored prospectively on a composite anastomotic-leak control rate; individual-grade comparisons (e.g. Grade C alone), stratified-subgroup analyses, and low-frequency secondary outcomes were prespecified as exploratory, are interpreted descriptively with 95% confidence intervals, and require confirmation in larger studies. Seventh, awareness of drain-fluid amylase values by treating clinicians is intrinsic to safe clinical care and could not be eliminated, although both arms were managed under identical, threshold-based drain-removal criteria. Finally, only patients with malignant disease undergoing open PD were enrolled; results may not extend to benign disease, chronic pancreatitis or minimally invasive PD.

### Future directions

Adequately sized, multicenter randomized validation with operator-independent application across heterogeneous surgical environments is the immediate priority. Other directions include: feasibility and effectiveness of Glubran^®^2 in laparoscopic and robotic pancreaticoduodenectomy; long-term assessment of anastomotic patency and pancreatic function; prospective cost-effectiveness analyses across healthcare systems; evaluation of Glubran^®^2 in distal pancreatectomy and pancreatic enucleation; and integration with risk-stratification models (FRS, a-FRS) and selective drain-management protocols [34].

## Conclusion

In this single-center randomized controlled trial of 100 patients undergoing open pancreaticoduodenectomy, reinforcement of the pancreaticojejunostomy with Glubran^®^2 was associated with a significantly lower rate of POPF (Grade B/C), shorter time to drain removal, lower overall morbidity, and shorter length of hospital stay, with an acceptable safety profile. These findings should be regarded as hypothesis-generating; confirmation in an adequately sized, multicenter randomized trial with operator-independent application of the adhesive is required before any recommendation for routine clinical adoption.

## Data Availability

The datasets utilzed & examined throughout the present research are accessible from the corresponding author on reasonable request.

## Abbreviations

a: FRS—alternative Fistula Risk Score
ASA: American Society of Anesthesiologists
BL: biochemical leak
BMI: body mass index
CCI: Comprehensive Complication Index
CDC: Centers for Disease Control and Prevention
CI: confidence interval
CR: POPF—clinically relevant postoperative pancreatic fistula
DFA: drain fluid amylase
DGE: delayed gastric emptying
ECOG: Eastern Cooperative Oncology Group
FRS: Fistula Risk Score (Callery)
HJ: hepaticojejunostomy
ICU: intensive care unit
ISGPS: International Study Group of Pancreatic Surgery
ITT: intention—to—treat
NNT: number needed to treat
OR: odds ratio
PD: pancreaticoduodenectomy
PJ: pancreaticojejunostomy
POD: postoperative day
POPF: postoperative pancreatic fistula
PP: per—protocol
PPH: post—pancreatectomy hemorrhage
RCT: randomized controlled trial
ULN: upper limit of normal

## References

[1] Winter JM, Cameron JL, Campbell KA, Arnold MA, Chang DC, Coleman J, et al. 1423 pancreaticoduodenectomies for pancreatic cancer: a single-institution experience. J Gastrointest Surg. 2006;10(9):1199–210. 17114007 10.1016/j.gassur.2006.08.018

[2] Bassi C, Marchegiani G, Dervenis C, Sarr M, Abu Hilal M, Adham M, et al. The 2016 update of the International Study Group (ISGPS) definition and grading of postoperative pancreatic fistula: 11 years after. Surgery. 2017;161(3):584–91. 28040257 10.1016/j.surg.2016.11.014

[3] Wente MN, Veit JA, Bassi C, Dervenis C, Fingerhut A, Gouma DJ, et al. Postpancreatectomy hemorrhage (PPH): an International Study Group of Pancreatic Surgery (ISGPS) definition. Surgery. 2007;142(1):20–5. 17629996 10.1016/j.surg.2007.02.001

[4] Shrikhande SV, Sivasanker M, Vollmer CM, Friess H, Besselink MG, Fingerhut A, et al. Pancreatic anastomosis after pancreatoduodenectomy: a position statement by the International Study Group of Pancreatic Surgery (ISGPS). Surgery. 2017;161(5):1221–34. 28027816 10.1016/j.surg.2016.11.021

[5] Cheng Y, Ye M, Xiong X, Peng S, Wu HM, Cheng N, et al. Fibrin sealants for the prevention of postoperative pancreatic fistula following pancreatic surgery. Cochrane Database Syst Rev. 2016;2:CD009621. 26876721 10.1002/14651858.CD009621.pub2

[6] Lillemoe KD, Cameron JL, Kim MP, Campbell KA, Sauter PK, Coleman JA, et al. Does fibrin glue sealant decrease the rate of pancreatic fistula after pancreaticoduodenectomy? Results of a prospective randomized trial. J Gastrointest Surg. 2004;8(7):766–72. 15531229 10.1016/j.gassur.2004.06.011

[7] Garcia CD, Ballester AM, Aliena-Valero A, Carabén-Redaño A, Llombart-Cussac A. Use of cyanoacrylate adhesives in general surgery. Surg Today. 2015;45(8):939–56. 25344231 10.1007/s00595-014-1056-4

[8] Montanaro L, Arciola CR, Cenni E, Ciapetti G, Savioli F, Filippini F, et al. Cytotoxicity, blood compatibility and antimicrobial activity of two cyanoacrylate glues for surgical use. Biomaterials. 2001;22(1):59–66. 11085384 10.1016/s0142-9612(00)00163-0

[9] Gaspar AF, Kemp R, Sankarankutty AK, Lopes Júnior JR, Filho JAF, Avezum VAPAF, et al. Influence on postoperative results and cost-effectiveness of using cyanoacrylate glue for pancreaticojejunal anastomosis after duodenopancreatectomy. Pancreas. 2025;54(7):e610–7. 40127250 10.1097/MPA.0000000000002479

[10] Probst P, Grummich K, Heger P, Zaschke S, Knebel P, Ulrich A, et al. Blinding in randomized controlled trials in general and abdominal surgery: protocol for a systematic review and empirical study. Langenbecks Arch Surg. 2019;404(3):273–84. 30824993 10.1007/s00423-019-01761-6

[11] Wente MN, Bassi C, Dervenis C, Fingerhut A, Gouma DJ, Izbicki JR, et al. Delayed gastric emptying (DGE) after pancreatic surgery: a suggested definition by the International Study Group of Pancreatic Surgery (ISGPS). Surgery. 2007;142(5):761–8. 17981197 10.1016/j.surg.2007.05.005

[12] Mangram AJ, Horan TC, Pearson ML, Silver LC, Jarvis WR. Guideline for prevention of surgical site infection, 1999. Centers for Disease Control and Prevention (CDC) Hospital Infection Control Practices Advisory Committee. Am J Infect Control. 1999;27(2):97–132. 10196487

[13] Dindo D, Demartines N, Clavien PA. Classification of surgical complications: a new proposal with evaluation in a cohort of 6336 patients and results of a survey. Ann Surg. 2004;240(2):205–13. 15273542 10.1097/01.sla.0000133083.54934.ae PMC1360123

[14] Barakat O, Ozaki CF, Wood RP. Topically applied 2-octyl cyanoacrylate (Dermabond) for prevention of postoperative pancreatic fistula after pancreaticoduodenectomy. J Gastrointest Surg. 2012;16(8):1499–507. 22580842 10.1007/s11605-012-1908-4

[15] Bassi C, Dervenis C, Butturini G, Fingerhut A, Yeo C, Izbicki J, et al. Postoperative pancreatic fistula: an International Study Group (ISGPF) definition. Surgery. 2005;138(1):8–13. 16003309 10.1016/j.surg.2005.05.001

[16] Tashiro S, Murata E, Hiraoka T, Miyauchi Y, Yamashita Y, Hamada E, et al. New technique for pancreaticojejunostomy using a biological adhesive. Br J Surg. 1987;74(5):392–4. 3594133 10.1002/bjs.1800740523

[17] Adelmeijer J, Porte RJ, Lisman T. In vitro effects of proteases in human pancreatic juice on stability of liquid and carrier-bound fibrin sealants. Br J Surg. 2013;100(11):1498–504. 24037572 10.1002/bjs.9243

[18] D’Andrea AA, Costantino V, Sperti C, Pedrazzoli S. Human fibrin sealant in pancreatic surgery: it is useful in preventing fistulas? A prospective randomized study. Ital J Gastroenterol. 1994;26(6):283–6. 7949264

[19] Kleespies A, Rentsch M, Seeliger H, Albertsmeier M, Jauch KW, Bruns CJ. Blumgart anastomosis for pancreaticojejunostomy minimizes severe complications after pancreatic head resection. Br J Surg. 2009;96(7):741–50. 19526614 10.1002/bjs.6634

[20] Peng SY, Wang JW, Lau WY, Cai XJ, Mou YP, Liu YB, et al. Conventional versus binding pancreaticojejunostomy after pancreaticoduodenectomy: a prospective randomized trial. Ann Surg. 2007;245(5):692–8. 17457161 10.1097/01.sla.0000255588.50964.5d PMC1877076

[21] Inthasotti W, Teepongkaruna S, Chaibut K, Lertsaranphibal U, Suksamak P. Incidence of pancreatic fistula after using N-butyl-2-cyanoacrylate glue for pancreaticojejunostomy anastomosis after pancreatoduodenectomy in Rajavithi Hospital. J Med Assoc Thai. 2018;101:S69–75.

[22] Bae JH, Kim GC, Ryeom HK, Shin HK, Kim SK, Choi YH, et al. Percutaneous embolization of persistent biliary and enteric fistulas with Histoacryl. J Vasc Interv Radiol. 2011;22(6):879–83. 21482136 10.1016/j.jvir.2011.01.453

[23] Molinari E, Bassi C, Salvia R, Butturini G, Crippa S, Talamini G, et al. Amylase value in drains after pancreatic resection as predictive factor of postoperative pancreatic fistula. Ann Surg. 2007;246(2):281–7. 17667507 10.1097/SLA.0b013e3180caa42f PMC1933557

[24] Dugalic VD, Knezevic DM, Obradovic VN, Lekic NS, Pavlovic IM, Milosavljevic TN. Drain amylase value as an early predictor of pancreatic fistula after cephalic duodenopancreatectomy. World J Gastroenterol. 2014;20(26):8691–9. 25024627 10.3748/wjg.v20.i26.8691 PMC4093722

[25] Kull S, Martinelli I, Briganti E, Losi P, Spiller D, Tonlorenzi S, et al. Glubran 2 surgical glue: in vitro evaluation of adhesive and mechanical properties. J Surg Res. 2009;157(1):e15–21. 19439320 10.1016/j.jss.2009.01.034

[26] Alfieri S, Di Miceli D, Pericoli Ridolfini M, Rotondi F, Quinto R, Doglietto GB. Glubran 2: a new acrylic glue for neuroendovascular, surgical and endoscopic applications. Interv Neuroradiol. 2001;7(Suppl 1):S269–71.

[27] Gong J, He S, Cheng Y, Cheng N, Gong J, Zeng Z. Fibrin sealants for the prevention of postoperative pancreatic fistula following pancreatic surgery. Cochrane Database Syst Rev. 2018;6:CD009621. 29934987 10.1002/14651858.CD009621.pub3 PMC6513198

[28] Probst P, Hüttner FJ, Klaiber U, Knebel P, Ulrich A, Büchler MW, et al. Evidence Map of Pancreatic Surgery — A living systematic review with meta-analyses by the International Study Group of Pancreatic Surgery (ISGPS). Surgery. 2021;170(5):1517–24. 34187695 10.1016/j.surg.2021.04.023

[29] Uijterwijk BA, Lemmers DH, Ghidini M, Wilmink JW, Dolan A, Davidson B, et al. The Five Periampullary Cancers, not Just Different Siblings but Different Families: An International Multicenter Cohort Study. Ann Surg Oncol. 2024;31(9):6157–69. 38888860 10.1245/s10434-024-15555-8

[30] Callery MP, Pratt WB, Kent TS, Chaikof EL, Vollmer CM Jr. A prospectively validated clinical risk score accurately predicts pancreatic fistula after pancreatoduodenectomy. J Am Coll Surg. 2013;216(1):1–14. 23122535 10.1016/j.jamcollsurg.2012.09.002

[31] Mungroop TH, van Rijssen LB, van Klaveren D, Smits FJ, van Woerden V, Linnemann RJ, et al. Alternative fistula risk score for pancreatoduodenectomy (a-FRS): design and international external validation. Ann Surg. 2019;269(5):937–43. 29240007 10.1097/SLA.0000000000002620

[32] Gaujoux S, Cortes A, Couvelard A, Noullet S, Clavel L, Rebours V, et al. Fatty pancreas and increased body mass index are risk factors of pancreatic fistula after pancreaticoduodenectomy. Surgery. 2010;148(1):15–23. 20138325 10.1016/j.surg.2009.12.005

[33] Bini A, Grazia M, Petrella F, Stella F, Massera F, Camplese P, et al. Clinical applications of Glubran 2: a synthetic glue in thoracic surgery. Ann Thorac Surg. 2003;75(1):330–1.

[34] McMillan MT, Malleo G, Bassi C, Allegrini V, Casetti L, Drebin JA, et al. Multicenter, prospective trial of selective drain management for pancreatoduodenectomy using risk stratification. Ann Surg. 2017;265(6):1209–18. 27280502 10.1097/SLA.0000000000001832

